# Optimization Correction Strength Using Contra Bending Technique without Anterior Release Procedure to Achieve Maximum Correction on Severe Adult Idiopathic Scoliosis

**DOI:** 10.1155/2016/7396853

**Published:** 2016-03-15

**Authors:** Ahmad Jabir Rahyussalim, Ifran Saleh, Dyah Purnaning, Tri Kurniawati

**Affiliations:** ^1^Department of Orthopaedics and Traumatology, Faculty of Medicine, University of Indonesia, Jakarta 10430, Indonesia; ^2^Stem Cell and Tissue Engineering Cluster, MERC Faculty of Medicine, University of Indonesia, Jakarta 10430, Indonesia

## Abstract

Adult scoliosis is defined as a spinal deformity in a skeletally mature patient with a Cobb angle of more than 10 degrees in the coronal plain. Posterior-only approach with rod and screw corrective manipulation to add strength of contra bending manipulation has correction achievement similar to that obtained by conventional combined anterior release and posterior approach. It also avoids the complications related to the thoracic approach. We reported a case of 25-year-old male adult idiopathic scoliosis with double curve. It consists of main thoracic curve of 150 degrees and lumbar curve of 89 degrees. His curve underwent direct contra bending posterior approach using rod and screw corrective manipulation technique to achieve optimal correction. After surgery the main thoracic Cobb angle becomes 83 degrees and lumbar Cobb angle becomes 40 degrees, with 5 days length of stay and less than 800 mL blood loss during surgery. There is no complaint at two months after surgery; he has already come back to normal activity with good functional activity.

## 1. Introduction 

Adult scoliosis is defined as a spinal deformity in a skeletally mature patient with a Cobb angle of more than 10 degrees in the coronal plain [1, 2]. Adult scoliosis can be separated into three major groups. The first is type 1, primary degenerative scoliosis, mostly on the basis of a disc and/or facet joint arthritis, affecting those structures asymmetrically with predominantly back pain symptoms, often accompanied with or without spinal stenosis (central as well as lateral stenosis); it is also classified as “de novo” scoliosis. Type 2 is idiopathic adult scoliosis of the thoracic and/or lumbar spine which progresses in adult life and is usually combined with secondary degeneration and/or imbalance. Some patients had no surgical treatment or surgical correction or fusion in adolescence in the thoracic or thoracolumbar spine. The last is type 3 which is divided into secondary adult curves in the context of an oblique pelvis, for example, due to a leg length discrepancy or hip pathology as well as a secondary curve in idiopathic, neuromuscular, and congenital scoliosis and also asymmetrical anomalies at the lumbosacral junction, and in the context of a metabolic bone disease (mostly osteoporosis) combined with asymmetric arthritic disease and/or vertebral fractures [1].

Progress in surgical techniques and technology of scoliosis treatment is significantly supported by progress in anesthesia for spinal surgery and by more sophisticated and precise diagnostic imaging and differentiated application of invasive and functional diagnostic tests, increased patient awareness, and the patient's unwillingness to accept their limitations and pains [1, 3]. In the past, the treatment of severe idiopathic scoliosis with the main curve more than 80 degrees was performed by an anterior release with an open thoracotomy [2, 3]. Nowadays, there were similar results between patients that were treated with an anterior release with an open thoracotomy to those who underwent posterior-only approach and avoided the negative effects on pulmonary function which the anterior release causes [2].

**Figure 1:**
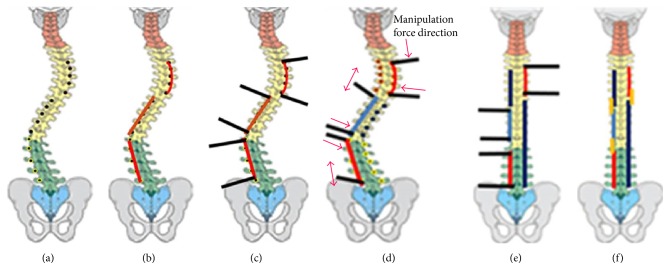
Rod and screw corrective manipulation technique procedure. (a) Pedicle screw placement. (b) Correction rod placement. (c) Correction bar placement. (d) Correction maneuver. (e) Correction rod placement. (f) Connecting rod placement.

Basically the difference between the rod and screw corrective manipulation approach and the previous one is the management of spinal column (Figure 1). Spinal column in the previous technique was attacked by anterior release and/or corpectomy procedure to weakened the column and make it easy to be manipulated. Meanwhile spinal column in this case with direct contra bending procedure was preserved. The previous technique has a lot of morbidity risks than the technique which was applied in this case.

## 2. Case Illustration

It was a 25-year-old man with complaint of crooked on his back 14 years ago. He did not seek any medical help because he did not feel any limitation in his daily activity. He went to traditional medicine provider and was treated there for several years.

**Figure 2:**
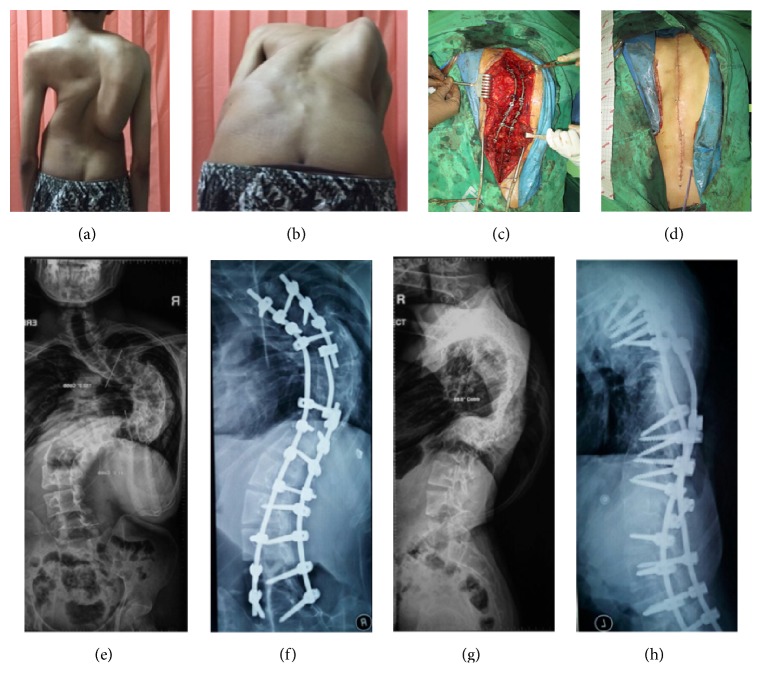
(a) and (b) Clinical picture on standing and forward bending position. It shows right rib hump and asymmetry of right and left back. (c) Intraoperative picture after rod and screw placement and manipulation. (d) Postoperative picture after suturing midline incision on back area. It shows asymmetry improvement of rib hump and balance. (e) Posterolateral view of cervicothoracolumbar spine before surgery manipulation. (f) Posterolateral view of thoracolumbar spine after surgery using rod and screw corrective manipulation technique. (g) Lateral view of cervicothoracolumbar spine before surgery manipulation. (h) Lateral view of thoracolumbar spine after RSCM technique surgery.

Physical examination showed right curvature of thoracic spine and right sided rib hump (Figures 2(a) and 2(b)). There was no cafe Au Lait. On palpation there was no muscle spasm nor pain. On the movement examination, the forward flexion was 0 to 70 degrees, the extension was 0 to 15 degrees, the left lateral bending was 0 to 20 degrees, and the right lateral bending 0 to 10 degrees (Table 1).

The radiograph imaging showed proximal thoracic, main thoracic, and lumbar curve. Proximal thoracic Cobb angle is 25 degrees, lower end vertebrae (LEV) is at T5, upper end vertebrae (UEV) is at T2, and the apex is at T4. Main thoracic Cobb angle is 150 degrees, lower end vertebrae (LEV) is at T11, upper end vertebrae (UEV) is at T4, and the apex is at T7, whereas the lumbar Cobb angle is 89 degrees, lower end vertebrae (LEV) is at L5, upper end vertebrae (UEV) is at T12, and the apex is at L3 (Figures 2(e) and 2(f)). The kyphosis angle is 89.5 degrees (Figures 2(g) and 2(h)).

It was diagnosed with adult idiopathic scoliosis Lenke 3B+ which meant that the scoliosis had double major curve, nonstructural curve type in proximal thoracic, and structural curve type of both main thoracic and lumbar. The central sacral vertical line touches the apical bodies and the thoracic sagittal profile was positive (hyperkyphotic, kyphosis curve > 40 degrees).

## 3. Direct Contra Bending Manipulation Technique

The surgical procedures have been performed by using direct contra bending manipulation.

The screw was set at thoracic 3 (T3) up to lumbar 5 (L5) (Figures 2(c) and 2(d)). The procedure was used by doing direct contra bending manipulation to the rods and screws which were implanted. Rod and screw placement were based on the deformity of each spinal segment according to the pathobiomechanics of deformity. Direct contra bending manipulation was performed to facilitate spinal correction into normal alignment in sagittal and coronal plane. Implants used in this procedure are pedicle screws, long rod, short rod, and connecting rod. Adequate pedicle screw placement becomes an absolute requirement for this direct contra bending manipulation because inadequate screw placement would lead screw pull out.

The principle of direct contra bending manipulation technique is the fact that we need higher energy or force to withstand resistance or the curve load in order to realign the bent spinal curve which can be obtained by two methods, namely, to decrease the curve resistance force or to increase the force to straighten the curve. In order to decrease the curve resistance force, soft tissue release or osteotomy procedure can be done, while to increase the realigning force the screws must be placed accurately into the pedicle, creating a linear segmental curve and increasing curve leverage power manually or using machine (robotic).

To optimize the direct contra bending manipulation, the spine is divided into three segments, the upper thoracic segment (T3–T7), the lower thoracic segment (T8–T12), and the lumbar segment (L1–L5) based on different thoracic and lumbar sagittal alignments. The thoracic segments are divided into two in order to split thoracic curve stiffness and facilitate the correction process.

## 4. Discussion

Scoliosis comes from a Greek word that means curvature, which implies pathologic conditions in the cervical, thoracic, or lumbar vertebrae that form the center of the column which is in the midline. Scoliosis is a spinal deformity that describes the disorder deviation and rotational vertebrae laterally. Scoliosis usually forms a curve (curve C) or two curves (curve S) [4, 5]. In this case, the patient had two curves in main thoracic and lumbar. The Cobb angle in main thoracic was 150 degrees and in lumbar it was 89 degrees. During the past decade, the attention has been increased to the problem of treating adult society with idiopathic scoliosis [6].

There are some indications if adult patient with scoliosis needs to be treated with surgery: pain, progression of the curve, and compromise of respiratory function, either alone or in combination. Precise conventional imaging and radiological procedures are very useful in rapid reconstruction of the spine vertically and in obtaining clear understanding of the pathology [1]. In this case we only performed conventional imaging with the result showing that the Cobb angle proximal thoracic Cobb was 25 degrees, lower end vertebrae (LEV) is at T5, upper end vertebrae (UEV) is at T2, and the apex is at T4. Main thoracic Cobb angle is 150 degrees, lower end vertebrae (LEV) is at T11, upper end vertebrae (UEV) is at T4, and the apex is at T7, whereas the lumbar Cobb angle was 89 degrees, lower end vertebrae (LEV) is at L5, upper end vertebrae (UEV) is at T12, and the apex is at L3. The kyphosis angle is 89.5 degrees.

Some surgery approaches can be divided into posterior approach, anterior approach, and combined procedures. Crostelli et al. conducted a study of 25 patients who underwent arthrodesis by posterior-only approach and instrumentation by all-pedicle screw [2]. They concluded that posterior spinal fusion with pedicle screw instrumentation will obtain good and stable correction for patients with severe scoliosis [7].

Two months after procedures, the patient was asked to complete and fill questionnaire SRS 24, to measure his quality of life (Table 2). It shows that the patient's SRS score is 89 out of 120, which is 74%. We conclude that from this questionnaire, the patient has good score based on SRS score questionnaire especially in self-image, function after surgery, and also function activity.

In our case, postoperatively the body height of the patient increased by 8 cm and sitting height of the patient increased by 5 cm, which is higher than the study conducted by spencer et al. [8] Adult scoliosis is a trending problem, which will arise in the future. The decision whether to do surgery or conservatively depends on the subjective and objective measures. Surgery approach in treating adult scoliosis consists of posterior, anterior, or combined approach, where posterior spinal fusion with pedicle screw-only instrumentations obtains good and stable correction of severe scoliosis (Figure 1).

The patients who were treated with direct contra bending manipulation technique presented superior results to those who underwent combined treatment and avoided the negative effects on pulmonary function that the anterior release caused. Posterior-only approach avoids the complications associated with thoracic approach and reduces the surgery time of combined procedure as well as morbidity and patient hospital recovery.

## 5. Conclusion

This case report presented the severe adult scoliosis that underwent single step direct contra bending manipulation technique and got better clinical and functional result in terms of the spinal column preservation and length of stay in hospital.

## Figures and Tables

**Table 1:** Physical examination parameters before and after surgery.

Parameter	Before surgery	After surgery	Improvement
Body height	149 cm	157 cm	8 cm
Sitting height	69 cm	77 cm	8 cm
Plumb line	1 cm (left)	2 cm (left)	1 cm shift left
Rib hump	7 cm (right)	5 cm (right)	2 cm
Shoulder tilt	0 cm	0 cm (right)	Balance
Pelvic tilt	0 cm	2 cm (right)	Tilting
Body-arm distance	4 cm (left)	3 cm (left)	1 cm

**Table 2:** SRS Questionnaire score after surgery.

SRS Questionnaire scores after surgery	Total patient score (maximal score)	Percent (100%, best; 20%, worst)
(1) Pain	26/(35)	74%
(2) General self-image	8/(15)	53%
(3) Self-image after surgery	12/(15)	80%
(4) Function after surgery	8/(10)	80%
(5) General function	11/(15)	73%
(6) Function activity	13/(15)	87%
(7) Satisfactory after surgery	11/(15)	73%
Total score	**89/(120)**	**74%**
